# Perioperative patient safety indicators and hospital surgical volumes

**DOI:** 10.1186/1756-0500-7-117

**Published:** 2014-02-28

**Authors:** Takefumi Kitazawa, Kunichika Matsumoto, Shigeru Fujita, Ai Yoshida, Shuhei Iida, Hirotoshi Nishizawa, Tomonori Hasegawa

**Affiliations:** 1 Department of Social Medicine, Toho University School of Medicine, 5-21-16, Omori-nishi, Ota-ku, Tokyo 143-8540, Japan; 2 All Japan Hospital Association, Sumitomo Fudosan Sarugaku-cho Building 7F, 2-8-8, Sarugaku-cho, Chiyoda-ku, Tokyo 101-8378, Japan; 3 Institute for Healthcare Quality Improvement, Tokyo Healthcare Foundation, 1-24-1, Asahigaoka, Nerima-ku, Tokyo 176-8530, Japan

**Keywords:** Patient safety indicators, Perioperative care, Observational study, Hospital surgical volume

## Abstract

**Background:**

Since the late 1990s, patient safety has been an important policy issue in developed countries. To evaluate the effectiveness of the activities of patient safety, it is necessary to quantitatively assess the incidence of adverse events by types of failure mode using tangible data. The purpose of this study is to calculate patient safety indicators (PSIs) using the Japanese Diagnosis Procedure Combination/per-diem payment system (DPC/PDPS) reimbursement data and to elucidate the relationship between perioperative PSIs and hospital surgical volume.

**Methods:**

DPC/PDPS data of the Medi-Target project managed by the All Japan Hospital Association were used. An observational study was conducted where PSIs were calculated using an algorithm proposed by the US Agency for Healthcare Research and Quality. We analyzed data of 1,383,872 patients from 188 hospitals who were discharged from January 2008 to December 2010.

**Results:**

Among 20 provider level PSIs, four PSIs (three perioperative PSIs and decubitus ulcer) and mortality rates of postoperative patients were related to surgical volume. Low-volume hospitals (less than 33rd percentiles surgical volume per month) had higher mortality rates (5.7%, 95% confidence interval (CI), 3.9% to 7.4%) than mid- (2.9%, 95% CI, 2.6% to 3.3%) or high-volume hospitals (2.7%, 95% CI, 2.5% to 2.9%). Low-volume hospitals had more deaths among surgical inpatients with serious treatable complications (38.5%, 95% CI, 33.7% to 43.2%) than high-volume hospitals (21.4%, 95% CI, 19.0% to 23.9%). Also Low-volume hospitals had lower proportion of difficult surgeries (54.9%, 95% CI, 50.1% to 59.8%) compared with high-volume hospitals (63.4%, 95% CI, 62.3% to 64.6%). In low-volume hospitals, limited experience may have led to insufficient care for postoperative complications.

**Conclusions:**

We demonstrated that PSIs can be calculated using DPC/PDPS data and perioperative PSIs were related to hospital surgical volume. Further investigations focusing on identifying risk factors for poor PSIs and effective support to these hospitals are needed.

## Background

Since the late 1990s, patient safety has been an important policy issue in developed countries. To evaluate the effectiveness of the activities of patient safety, it is necessary to quantitatively assess the incidence of adverse events by types of failure mode using tangible data. However, the conventional retrospective clinical record reviews are now difficult to conduct because of the amount of effort required and the stringent regulations regarding the protection of personal information [1]. To develop alternative method to measure the incidence of adverse events is an important issue, although claim data or hospital administrative data have been used for this purpose [2,3].

The US Agency for Healthcare Research and Quality (AHRQ) started developing clinical indicators in the early 1990s, known as AHRQ quality indicators (QIs) [4]. AHRQ QIs are categorized on the basis of the domain in healthcare that they reflect. Among them, patient safety indicators (PSIs) are a set of QIs that provide information regarding potentially preventable in-hospital complications and adverse events following surgeries, procedures, and childbirth. PSIs comprise seven regional level indicators and 20 provider level (hospital level) indicators.

Each PSI has a denominator and numerator that are defined using ICD-9-CM and DRG codes. Both ICD-9-CM and DRG codes are widely used for not only hospital reimbursement and monitoring of health services but also objectively measure patient safety concerns in a hospital on the basis of information collected daily from discharged patients. PSIs focus on potentially preventable complications, and they use information of diagnoses made after surgeries or procedures [5]. PSIs have been validated using data obtained from Veterans Affairs hospitals [6-8] and are currently being used to reimburse hospitals. The US Center for Medicare and Medicaid Services adopted PSIs in value-based purchasing programs to measure hospital performance [9]. AHRQ has developed and released a user’s guide and software for calculating PSIs.

In Japan, the Diagnosis Procedure Combination/per-diem payment system (DPC/PDPS) was introduced in 2003 to reimburse university hospitals and is now a standard reimbursement system for acute care hospitals. DPC/PDPS reimbursement data include electronic information regarding diagnoses and details of daily medical procedures, surgeries, and prescribed medicines as well as patient demographics. DPC/PDPS data use ICD-10 codes for diagnoses and K-codes (Japanese original codes) for surgeries. DPC/PDPS data can be used to improve healthcare transparency and quality. For example, the All Japan Hospital Association (AJHA), one of the largest nation-wide hospital associations comprising 2,300 hospitals, handles the administration of the Medi-Target project, a benchmark project using clinical indicators based on DPC/PDPS data. Participation in Medi-Target project was optional, and there were 27 participating hospitals in 2008, 174 hospitals in 2009, and 182 hospitals in 2010.

The purpose of the present study was to calculate PSIs using DPC/PDPS data and to investigate the incidence of adverse events by type. The relationship among hospital surgical volume, difficulty of surgeries, and perioperative PSIs were also investigated.

## Methods

DPC/PDPS data of the Medi-Target project from January 2008 to December 2010 were used for analysis. PSI technical specifications versions 4.2 released by AHRQ were used for calculating PSIs.

Technical specifications designate both inclusion and exclusion criteria for the numerators and denominators of each PSI with patient demographics such as age, length of hospital stay, primary and secondary diagnoses, surgeries, and procedures defined by ICD-9-CM codes. In this study, ICD-9-CM codes were converted to ICD-10 codes and ICD-9-CM procedure codes were converted to K-codes using a translation table released by the Medical Information System Development Center of Japan. Thereafter, we constructed a database storing information regarding patient demographics, primary and secondary diagnoses, comorbidities at admission, postadmission complications, surgeries, and procedures.

To investigate the relationship between hospital surgical volume and PSIs, we divided the hospitals into three groups on the basis of volume, i.e., high-volume, mid-volume, and low-volume, using the 66th and 33rd percentiles of the distribution of surgeries per month. Surgical difficulty and patient severity were also considered. The Association of Social Insurance Committees of the Surgical Societies of Japan classified surgeries into five classes on the basis of difficulty, i.e., A–E with E being the most difficult. Classes D and E were defined as surgeries that should be performed by surgeons with more than 15 years of experience and special skills in the area.

We assessed patient severity using the Charlson Comorbidity Index [10] on the basis of comorbidities at admission. The Charlson Comorbidity Index originally used ICD-9-CM codes and has been translated to ICD-10 codes using an algorithm proposed by Quan et al. [11].

Analysis of variance (ANOVA) was used for statistical analysis among high-, mid- and low-volume hospitals. Multiple liner regression analysis with the forced entry method was used to determine the relationship between surgical volume and PSIs adjusting for patient age, severity, and proportion of difficult surgeries. We considered surgical difficulty as one of the characteristics of surgery and that variable was put into multiple linear regression analysis. SPSS version 20 was used for statistical analysis and a p value <0.05 was considered statistically significant.

This study used only anonymised data, and did not use human or animals. In Japan, for this kind of study no institutional review is requested [12].

## Results

In the present analysis, we used data of 1,383,872 patients treated at 188 hospitals. The mean age of the patients was 61.5 years and the average length of hospital stay was 16.2 days. Basic hospital characteristics are shown in Table 1. There were 587,506 operative patients, and they were used for further analyses. We accounted for the correlation of data within hospitals. We have observed a correlation between number of surgical operations per month and number of patients discharged per month. The correlation coefficient of this relationship was over 0.9 and that was statistically significant (p < 0.01). Low-volume hospitals treated more elderly and female patients, and also reported longer hospital stays and higher mortality rates than mid- or high-volume hospitals. High-volume hospitals performed more difficult surgeries than low-volume hospitals. The mortality rate of postoperative patients was also related to surgical volume: low-volume hospitals, 5.7%; mid-volume hospitals, 2.9%; and high-volume hospitals, 2.7% (p < 0.01 by ANOVA).

**Table 1:** Characteristics of hospitals

		**Low-volume**		**Mid-volume**		**High-volume**		**Total**	***p *** **value†**
	**Mean**	**S.D.**	**Mean**	**S.D.**	**Mean**	**S.D.**	**Mean**	**S.D.**	
Patient level characteristics									
Age (year)	68.3	5.8	59.1	12.8	57.3	4.7	61.5	9.8	*p* < 0.05
Proportion of females (%)	50.3	9.8	46.4	9.4	45.8	3.5	47.5	8.2	*p* < 0.01
Charlson comorbidity index	1.10	0.66	1.04	0.46	1.08	0.32	1.07	0.49	n.s.
Length of hospital stay (days)	18.9	6.2	15.5	4.0	14.2	1.7	16.2	4.7	*p* < 0.01
Hospital level characteristics									
Number of discharged patients per month	141.4	66.8	336.7	86.6	693.7	219.1	393.8	270.3	*p* < 0.01
Number of surgeries per month	45.1	24.5	135.2	30.2	313.3	114.5	166.1	132.3	*p* < 0.01
Percent of surgical patients (%)	31.2	12.4	41.4	8.8	45.4	7.5	39.4	11.4	*p* < 0.01
Proportion of difficult surgeries (%)	54.9	19.1	60.3	10.3	63.4	4.4	59.6	13.1	*p* < 0.05
Mortality rates (%)	6.1	2.9	4.8	2.0	4.1	1.5	5.0	2.3	*p* < 0.01
Mortality rates of postoperative patients (%)	5.7	6.8	2.9	1.4	2.7	0.9	3.7	3.7	*p* < 0.01

†ANOVA was used for statistical analysis and Scheffe’s test for post hoc comparisons.

The relationship between hospital surgical volume and PSIs is shown in Table 2. High-volume hospitals had higher scores for PSI#9 (postoperative hemorrhage or hematoma) and PSI#13 (postoperative sepsis). Low-volume hospitals had higher scores for PSI#3 (pressure ulcer) and PSI#4 (death among surgical inpatients with serious treatable complications). Low-volume hospitals incurred more deaths among surgical inpatients with serious treatable complications compared with high-volume hospitals, although the proportion of difficult surgeries (classes E and D) was less than that of high-volume hospitals.

**Table 2:** Relationship between surgical volume and PSIs

		**n**	**Low-volume**	**Mid-volume**	**High-volume**	***p *** **value†**
PSI#2	Death in low-mortality DRGs*	151,447	0.0 (0.0-0.0)	0.0 (0.0-0.0)	0.0 (0.0-0.0)	n.s.
PSI#3	Decubitus ulcer	998,048	8.0 (5.1-10.9)	4.5 (3.5-5.5)	3.8 (3.0-4.6)	*p* < 0.05
PSI#4	Death among surgical inpatients with serious treatable complications	28,314	384.6 (337.4-431.8)	293.3 (249.8-336.8)	214.5 (190.4-238.6)	*p* < 0.01
PSI#5	Foreign body left during procedure	1,220,717	0.0 (0.0-0.0)	0.0 (0.0-0.0)	0.0 (0.0-0.0)	n.s.
PSI#6	Iatrogenic pneumothorax	1,170,714	0.0 (0.0-0.1)	0.1 (0.0-0.1)	0.1 (0.0-0.1)	n.s.
PSI#7	Central venous catheter-related blood stream infections	827,855	0.2 (0.1-0.3)	0.2 (0.1-0.3)	0.3 (0.0-0.6)	n.s.
PSI#8	Postoperative hip fracture	491,103	1.1 (0.0-2.1)	0.4 (0.2-0.5)	0.6 (0.0-1.2)	n.s.
PSI#9	Postoperative hemorrhage or hematoma	539,515	18.4 (6.8-30.0)	31.2 (15.4-47.1)	41.4 (33.8-49.0)	*p* < 0.05
PSI#10	Postoperative physiologic and metabolic derangement	532,175	3.3 (2.3-4.4)	2.6 (2.1-3.2)	3.6 (3.0-4.1)	n.s.
PSI#11	Postoperative respiratory failure	448,338	12.4 (1.4-23.5)	8.8 (5.4-12.2)	5.3 (3.2-7.3)	n.s.
PSI#12	Postoperative pulmonary embolism or deep vein thrombosis	537,366	5.3 (1.6-9.1)	4.9 (1.1-8.6)	7.3 (1.5-13.0)	n.s.
PSI#13	Postoperative sepsis	561,102	4.4 (2.5-6.4)	5.1 (3.9-6.3)	10.5 (8.1-13.0)	*p* < 0.01
PSI#14	Postoperative wound dehiscence	81,364	10.2 (4.6-15.8)	11.5 (8.3-14.7)	9.9 (8.1-11.7)	n.s.
PSI#15	Accidental puncture or laceration	1,185,502	0.0 (0.0-0.0)	0.0 (0.0-0.0)	0.0 (0.0-0.0)	n.s.
PSI#16	Transfusion reaction	1,220,717	0.0 (0.0-0.0)	0.0 (0.0-0.0)	0.0 (0.0-0.0)	n.s.
PSI#17	Birth trauma - injury to neonate	3,068	0.0 (0.0-0.0)	0.0 (0.0-0.0)	0.0 (0.0-0.0)	n.s.
PSI#18	Obstetric trauma - vaginal delivery with instruments	207	0.0 (0.0-0.0)	9.2 (3.6-15.8)	19.2 (7.2-33.7)	n.s.
PSI#19	Obstetric trauma - vaginal delivery without Instruments	1,184	0.0 (0.0-0.0)	12.5 (6.2-20.5)	21.7 (9.6-37.0)	n.s.

Score of PSIs are indicated by rate per 1,000 discharges. PSI#2 was calculated per 100, PSI#5 and #16 were observed cases.

Directly calculated 95% CI of PSI#18 and #19 had wide range and lower limit was negative number. To improve this situation, we performed bootstrap method to calculate these 95% CI.

PSI#1 and #20 were deleted since AHRQ quitted these support.

*In this study, we calculated Death in Low-Mortality DPC/PDPS. †ANOVA was used for statistical analysis and Scheffe’s test for post hoc comparisons.

Multiple linear regression analysis was performed to determine the relationship between surgical volume and three PSIs (PSI#4, #9, and #13), adjusting for patient age, severity, and proportion of difficult surgeries (Table 3). A significant relationship was observed between surgical volume and the three PSIs. Each additional surgical patient per month is associated with 0.2 fewer cases of Death among surgical inpatients with serious treatable complications /1,000 hospitalizations but 0.2 more cases of postoperative hemorrhage or hematoma and 0.3 more cases of postoperative sepsis/1,000 hospitalizations.

**Table 3:** Result of multiple linear regression analysis

	**PSI#4**		**PSI#9**		**PSI#13**		**Postoperative death**	
	**R**^**2**^	**p value**	**R**^**2**^	**p value**	**R**^**2**^	**p value**	**R**^**2**^	**p value**
	0.365	*p* < 0.01	0.129	*p* < 0.01	0.111	*p* < 0.01	0.406	*p* < 0.01
Valuables	Coefficient	p value	Coefficient	p value	Coefficient	p value	Coefficient	p value
Intercept		n.s.		*p* < 0.01		n.s.		*p < 0.05*
Age	0.374	*p* < 0.01	-0.229	*p* < 0.01	0.018	n.s.	0.183	*p* < 0.01
Charlson comorbidity index	0.160	*p* < 0.01	0.116	n.s.	0.106	n.s.	0.285	*p* < 0.01
Difficulty of surgery	-0.233	*p* < 0.01	-0.152	*p* < 0.05	0.016	n.s.	-0.437	*p* < 0.01
Surgical volume	-0.191	*p* < 0.01	0.168	*p* < 0.05	0.314	*p* < 0.01	-0.065	n.s.

Multiple linear regression analysis was used for statistical analysis.

## Discussion

The results of our study suggest that surgical volume was related to PSI#4, #9, and #13. In low-volume hospitals, experience to perform difficult surgeries was limited and they may have experienced difficulty in dealing with postoperative complications, thereby resulting in higher mortality rates. In high-volume hospitals, the proportion of difficult surgeries and surgical patients were high and their ability to detect complications, such as postoperative hemorrhage/hematoma or sepsis, may have been high, leading to the higher prevalence of postoperative complications. However, the experience of the hospital staff and greater resources to deal with complications may explain the relatively low mortality rates.

In the US, analysis of Nationwide Inpatient Sample Discharge Database suggested that high-volume hospitals had lower mortality rates among surgical inpatients with serious treatable complications who underwent coronary artery bypass grafting and Roux-en-Y gastric bypass surgeries [13].

PSIs are also used in other countries. For example, in the UK, PSIs are calculated using the AHRQ algorithm [14]. The Health Care Quality Indicators project conducted by the Organization for Economic Co-operation and Development developed an international benchmark system that included 12 PSIs among 59 candidate indicators [15]. In the present study, we demonstrated that PSIs can be calculated from DPC/PDPS data, which are easy to obtain and may be useful for international PSI comparisons.

Most previous Japanese studies regarding perioperative and operative patient safety focused on specific surgical procedures, such as cardiovascular and gastric cancer surgeries, using operative patient registration system [16]. DPC/PDPS is a standard reimbursement system for inpatients in acute care hospitals in Japan and contains electronic data regarding diagnoses, details of daily medical procedures, surgeries, and prescribed medications in addition to patient demographics.

This study is not free from limitations. First is the process of translation and difference of coding habits among countries. Some of the ICD-9-CM codes were difficult to translate to the DPC/PDPS codes. In Japan, some codes such as a foreign body left during procedure are seldom used. They might affect calculations, and we should be prudent in comparing results from different countries. We believe that there is a need to consider the next study to clarify the characteristics related to the translation of the technical specification, including conversion from ICD-9-CM to ICD-10 and the effect of study results. DPC/PDPS system has basically designed for reimbursement and incident reporting system has been managed separate from reimbursement system. There is possibility that information about the disease which developed after admission which do not affect the reimbursement would not be input to DPC/PDPC database. This cording habit might lead underestimate of PSIs. Second is representativeness of the dataset used in our study. In 2012, 1,496 acute care hospitals are reimbursed using DPC/PDPS all over Japan, the dataset of this study reflects about 10-15% of the DPC/PDPS hospitals, and they are not selected randomly. Participation in Medi-Target project is optional, and participating hospitals might pay more attention to quality and safety issues than non-participating hospitals. PSIs calculated in the study might not be applied to the other hospitals.

In the future, DPC/PDPS will be extended to reimburse outpatients. Although we used a dataset of hospitals participating in the Medi-Target project, PSIs of all acute care hospitals in Japan can be calculated without any special preparation using the method developed in this study. This is the first study to use large data, and further studies focusing on organizational safety issues using DPC/PDPS data are needed.

## Conclusions

In the present study, we demonstrated that DPC/PDPS data can be used to calculate PSIs. Perioperative PSIs were related to hospital surgical volume. The mortality rate of patients with operations was also related to surgical volume, and low-volume hospitals had higher mortality rates than mid- or high-volume hospitals. Low-volume hospitals had more deaths among surgical inpatients with serious treatable complications compared with high-volume hospitals, although the proportion of difficult surgeries (classes E and D) was less than that of high-volume hospitals. In low-volume hospitals, limited experience may have led to insufficient care of postoperative complications. Thus, effective support should be investigated focusing on these hospitals.

## Abbreviations

AJHA: All Japan Hospital Association; AHRQ: Agency for Healthcare Research and Quality; ANOVA: Analysis of variance; DPC/PDPS: Diagnosis procedure combination/per-diem payment system; PSIs: Patient safety indicators; QIs: Quality indicators.

## Competing interests

The authors declare that they have no competing interests.

## Authors’ contributions

TK participated in the design of the study, performed the data collection and the analysis, and drafted the manuscript. KM participated in the design of the study and performed the analysis. SF, AY, SI and HN performed the data collection and the analysis. TH conceived of the study, and participated in its design and helped to draft the manuscript. All authors read and approved the final manuscript.
